# Polarized RPE Secretome Preserves Photoreceptors in Retinal Dystrophic RCS Rats

**DOI:** 10.3390/cells12131689

**Published:** 2023-06-22

**Authors:** Kabir Ahluwalia, Juan-Carlos Martinez-Camarillo, Biju B. Thomas, Aditya Naik, Alejandra Gonzalez-Calle, Dimitrios Pollalis, Jane Lebkowski, Sun Young Lee, Debbie Mitra, Stan G. Louie, Mark S. Humayun

**Affiliations:** 1 Mann School of Pharmacy and Pharmaceutical Sciences, University of Southern California, Los Angeles, CA 90089, USA; kahluwal@usc.edu (K.A.); aanaik@usc.edu (A.N.); 2 USC Ginsburg Institute of for Biomedical Therapeutics, University of Southern California, Los Angeles, CA 90033, USA; juan.martinez@med.usc.edu (J.-C.M.-C.); biju.thomas@med.usc.edu (B.B.T.); gonz762@usc.edu (A.G.-C.); pollalis@usc.edu (D.P.); sunyoung.lee@med.usc.edu (S.Y.L.); debbiemitrax@gmail.com (D.M.); 3 USC Roski Eye Institute, Department of Ophthalmology, Keck School of Medicine, University of Southern California, Los Angeles, CA 90033, USA; 4 Regenerative Patch Technologies LLC, Menlo Park, CA 94028, USA; jane@regenerativepatch.com; 5 Department of Physiology & Neuroscience, Keck School of Medicine, University of Southern California, Los Angeles, CA 90089, USA

**Keywords:** retina, age-related macular degeneration, retinitis pigmentosa, photoreceptor, retinal pigmented epithelium, retinal degeneration, secretome, Royal College of Surgeons rat

## Abstract

Retinal degenerative diseases, including age-related macular degeneration (AMD) and retinitis pigmentosa, lack effective therapies. Conventional monotherapeutic approaches fail to target the multiple affected pathways in retinal degeneration. However, the retinal pigment epithelium (RPE) secretes several neurotrophic factors addressing diverse cellular pathways, potentially preserving photoreceptors. This study explored human embryonic stem cell-derived, polarized RPE soluble factors (PRPE-SF) as a combination treatment for retinal degeneration. PRPE-SF promoted retinal progenitor cell survival, reduced oxidative stress in ARPE-19 cells, and demonstrated critical antioxidant and anti-inflammatory effects for preventing retinal degeneration in the Royal College of Surgeons (RCS) rat model. Importantly, PRPE-SF treatment preserved retinal structure and scotopic b-wave amplitudes, suggesting therapeutic potential for delaying retinal degeneration. PRPE-SF is uniquely produced using biomimetic membranes for RPE polarization and maturation, promoting a protective RPE secretome phenotype. Additionally, PRPE-SF is produced without animal serum to avoid immunogenicity in future clinical development. Lastly, PRPE-SF is a combination of neurotrophic factors, potentially ameliorating multiple dysfunctions in retinal degenerations. In conclusion, PRPE-SF offers a promising therapeutic candidate for retinal degenerative diseases, advancing the development of effective therapeutic strategies for these debilitating conditions.

## 1. Introduction

Retinal dystrophies, such as age-related macular degeneration (AMD), are the leading cause of vision loss in industrialized countries. Globally, AMD is projected to reach 288 million cases by 2040 [1]. The molecular pathogenesis of AMD includes the accumulation of cellular debris and oxidative stress triggering chronic inflammation, retinal cell atrophy, and ultimately vision loss [2,3]. Currently, anti-vascular endothelial growth factor (VEGF) treatment is used for neovascular AMD (nAMD), which accounts for 10–15% of AMD cases. Until recently, there was no approved treatment for dry AMD (dAMD) and the only options to slow disease progression were dietary supplements high in antioxidants [4,5,6,7,8]. The first intraocular administration of a complement inhibitor (i.e., C3 inhibitor: pegcetacoplan) was approved by the FDA in February 2023 for the treatment of geographic atrophy (GA), the advanced form of dAMD. Although pegcetacoplan can ameliorate GA progression, it is unable to halt or reverse vision loss [9].

Due to the complex etiology of dAMD, investigational modalities have included antioxidants, visual cycle modulators, mitochondrial modulators, anti-inflammatory agents, complement inhibitors, neuroprotective agents, and stem cell therapies [10]. Limitations associated with monotherapeutic approaches are that they are unable to fully address the multiple pathways promoting AMD development, which may account for limited effectiveness. Stem cell-based therapies directly target GA and other retinal degenerative diseases by replacing atrophic tissue [11,12,13,14]. CPCB-RPE1, a subretinal implant containing polarized human embryonic stem cell (hESC)-derived retinal pigment epithelium (RPE) grown on ultrathin parylene membranes was implanted in GA patients in a phase I/IIa clinical trial and showed improvement in visual acuity [12,15,16].

The RPE has long been speculated as a source of various neuroprotective factors. Characterization of the RPE secretome has identified several proteins with neuroprotective properties in retinal degeneration models [17,18,19,20,21,22,23]. Unfortunately, the administration of these purified components has not led to an effective therapy in human trials [24,25]. During the development of CPCB-RPE1, photoreceptor preservation was seen beyond the borders of the implant [26]. These findings suggest that the RPE secretes factors capable of promoting neuronal survival [18,27,28,29,30,31,32,33], and we propose to study the RPE secretome as a combination therapy for retinal degenerative conditions.

Oxidative stress and inflammation are central to AMD pathogenesis, and it has been shown they significantly alter the RPE secretome, resulting in increased secretion of pro-angiogenic factors and decreased anti-inflammatory factors including complement factor H (CFH), a critical inhibitor of complement activation and inflammation, and the strongest genetic risk for AMD [34,35,36,37,38,39]. Following the approval of pegcetacoplan, it can be surmised that the RPE secretome, containing a complement inhibitor, is directly linked to disease pathology and treatment. Oxidative damage to the RPE secretome results in a hostile microenvironment, and we hypothesize that the enriched secretome from healthy hESC-RPE can restore the retinal microenvironment and promote photoreceptor preservation in retinal degeneration models. In this report, we demonstrate that polarized RPE soluble factors (PRPE-SF), specifically concentrated PRPE-SF (SF3), promote fetal retinal progenitor cell (fRPC) viability and survival. SF3 also delayed retinal degeneration in the Royal College of Surgeons (RCS) rat model, which was associated with reduced oxidative stress and inflammation. These findings support the conclusion that SF3 can alter the diseased retinal microenvironment, promoting preservation of photoreceptors and visual function, offering an alternative therapeutic strategy for AMD and other complex retinopathies.

## 2. Materials and Methods

### 2.1. hESC-RPE Cell Culture and PRPE-SF1 Production

**Figure 1:**
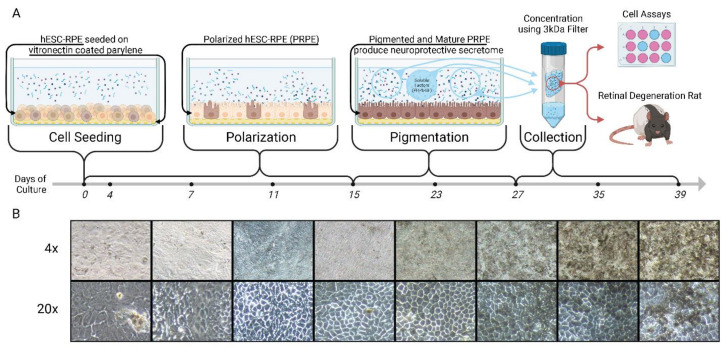
Diagram and timeline of polarized retinal pigmented epithelial soluble factor (PRPE-SF) production (**A**) with corresponding 4× and 20× images of PRPE (**B**). Human embryonic stem cells (hESC) differentiated into RPE were plated onto vitronectin-coated ultrathin parylene membranes and incubated in XVIVO10 medium. Significant morphological changes occurred during the first 15 days of cell culture resulting in polarization and the cobblestone shape expected of mature RPE. In the following 12 days there is was a rapid increase in pigmentation signifying the maturation of the hESC-RPE cells. PRPE-SF (SF1) was collected, concentrated (SF3) using a 3 kDa filter, and stored at −80 °C on days 27, 31, 35, and 39. Subsequently, SF1 and SF3 were characterized in cells and the Royal College of Surgeons rat model of retinal degeneration. Created with BioRender.com (accessed on 15 June 2023).

PRPE-SF1 was produced at USC using hESC-RPE cells designated as an intermediate cell bank (ICB), manufactured at the City of Hope under cGMP using the methods established by the CPCB [40,41,42,43,44]. The culture apparatus and PRPE-SF1 production scheme can be found in Figure 1. Briefly, H9 hESCs were expanded in mTeSR1 medium (Stemcell Technologies, Vancouver, BC, Canada), then the medium was replaced with XVIVO10 medium (Lonza, Basel, Switzerland) for 12 weeks to drive spontaneous RPE cell differentiation. Pigmented RPE-like cells were mechanically isolated followed by dissociation with TrypLE (Life Technologies, Grand Island, NY, USA). RPE-like cells were cultured on human vitronectin (AMS Biotechnology, Lake Forest, CA, USA)-coated plates with XVIVO10 medium and frozen at passage two. hESC-derived RPE cells were seeded onto vitronectin (Corning, Glendale, AZ, USA)-coated 10 mm × 20 mm parylene C membrane similar to the CPCB-RPE1 implant. The parylene C membrane has identical characteristics to CPCB-RPE1 as it is specially designed to mimic the Bruch’s membrane with a 0.3 µm thickness supported on a 6.0 µm thick mesh frame with diffusion regions for small molecules (manufactured at Leap Biomed Innovators, LLC, Pasadena, CA, USA) [41]. Parylene membranes were placed into 4-chamber wells (Corning Inc., Corning, NY, USA) prior to hESC-RPE seeding at a final seeding density of 1.43 × 10^5^ cells/cm^2^ and maintained at 37 °C in a 5% CO_2_ incubator. The medium was exchanged every 4 days, PRPE-SF was collected every 4 days starting on day 28 until day 40. The collected PRPE-SF was pooled and filtered with a 0.2 µm syringe filter system followed by 3-fold (3×) concentration, by volume, using Amicon centrifugal filter devices (Millipore Sigma, Burlington, MA, USA) with a 3 kD Ultracel regenerated cellulose membrane. Both unconcentrated (SF1) and concentrated (SF3) PRPE-SF were stored at −80 °C until use. Secreted proteins were characterized using a quantitative 40 human growth factor array Q1 (RayBiotech Inc., Nacross, GA, USA), and PEDF and LIF were determined by ELISA (Boster Biological Technology, Pleasanton, CA, USA).

### 2.2. Fetal Retinal Progenitor Cell (RPC) and ARPE-19 Culture

The biological activity of SF1 was characterized by using 18–20 weeks gestation of human fRPC. Fetal donor eyes were obtained from Lion Eye Institute under appropriate USC investigational review board approval. Following dissection, the retina was rinsed with PBS and broken up by passing the cells through a 27-gauge needle. The cells were washed with RPC media (DMEM/F12 [1:1], with 10% knockout replacement serum (KRS; Invitrogen), 1 N2 supplement (Invitrogen), 1 B-27 (Invitrogen), 20 ng/mL FGF2 (R&D Systems), and 20 ng/mL EGF (R&D Systems)). Cells were plated into Matrigel-coated plates and cultured for 24 h in RPC media then replaced with experimental media. After 24 h, plates were used in cell viability assays, apoptosis assays, immunocytochemistry staining, and RT-qPCR.

ARPE-19 cells were purchased from American Type Culture Collection (ATCC, Manassas, VA, USA) and were maintained in culture media composed of DMEM/F-12 (Corning Life Sciences, Corning, NY, USA) and 10% fetal bovine serum. Cell cultures were maintained at 37 °C in a 5% CO_2_ incubator. ARPE-19 cell assays are described below.

### 2.3. ARPE-19 Cell Assays

The 2′,7′ –dichlorofluorescin diacetate (DCFDA) assay is an intracellular assay for non-selectively detecting ROS. ARPE-19 cells were seeded into 96-well plates at a density of 1.25 × 10^5^ cells/cm^2^ in maintenance media. Following 24 h, cells were washed with HBSS (Cytiva, Marlborough, MA, USA) and the medium was replaced with experimental medium diluted 1:1 with fresh XVIVO10. After 24 h, cells were washed with HBSS and media replaced with 25 µM DCFDA (Sigma-Aldrich, St. Louis, MO, USA) in HBSS, and then placed in a CO_2_ incubator for 45 min prior to measuring fluorescence on a Biotek Synergy H1 microplate reader (BioTek, Winooski, VT, USA) using 485 nm excitation and 535 nm emission wavelengths. Percent change was calculated from blank-subtracted data using cells in HBSS with no DCFDA. Similarly, ARPE-19 cells were treated in 24-well plates (1.25 × 10^5^ cells/cm^2^) for 24 h, RNA was extracted as described below, and ROS-generating and -eliminating genes were probed.

### 2.4. Rhodopsin Staining

Following treatment and media removal, slides were fixed using 10% neutral buffered formalin for 10 min. Heat-induced antigen retrieval was performed with pH 6.0 citrate buffer. Samples were blocked with 5% BSA with 0.1% Triton X-100 for 30 min at room temperature (RT). Rhodopsin antibody was diluted 1:400 with 5% BSA and incubated overnight at 4 °C. Secondary antibody was diluted 1:100 with 5% BSA and incubated for 1 h at RT. Coverslips were mounted with 2 drops of fluorescent enhance mounting medium with DAPI (VECTASHIELD HardSet, Vector Labs, Newark, CA, USA). Slides were imaged on Keyence BZ-X700 Microscope (Keyence, Osaka, Japan).

### 2.5. TUNEL Assay

Following incubation with the various treatments, apoptosis assays were performed using Promega’s DeadEnd™ fluorometric TUNEL system (Promega, Madison, WI, USA) according to the manufacturer’s protocol. The reaction mix consisted of 45 µL of equilibration buffer, 5 µL nucleotide mix, and 1 µL rTdT enzyme, incubated at 37 °C for 1 h protected from light. The nuclear counterstain utilized 1 µg/mL propidium iodide in PBS for 15 min at RT protected from light. Wells were then washed 3 times with deionized water and replaced with PBS for imaging. The plate was then imaged using a Keyence BZ-X700 microscope (Keyence, Osaka, Japan).

### 2.6. RNA-Extraction and RT-qPCR

Following treatment, total RNA was isolated using a RNeasy Mini Kit (QIAGEN, Hilden, Germany) following the manufacturer’s protocol. RNA concentration and purity were assessed using a NanoDrop™ One (Thermo Fisher Scientific, Waltham, MA, USA). Reverse transcription was performed using a reverse transcription system (Promega, Madison, WI, USA). In this process, 500 ng of total RNA was used for transcription and the other components of the reaction mix were as described in the manufacturer’s protocol. RT-qPCR was performed using 10 ng cDNA, 300 nM forward and reverse primers, and 1× PowerUp SYBR Green Master Mix (Thermo Fisher Scientific, Waltham, MA, USA) in a 10 µL reaction. Forward and reverse primers were designed by Sigma-Aldrich (Supplementary Table S1) for fRPC studies. ARPE-19 primers were designed using Primer-Blast from NCBI and sequences are listed in Supplementary Table S2. RT-qPCR was performed on an Applied Biosystems Quantstudio 12 K Flex (Thermo Fisher Scientific, Waltham, MA, USA) with SYBR fluorescence with ROX passive reference. The thermal cycling conditions followed the standard cycling for primers with melting temperature greater than 60 °C: UDG activation for 2 min at 50 °C, dual-lock DNA polymerase for 2 min at 95 °C, followed by 40 cycles of denature for 15 s at 95 °C then annealing/extending for 1 min at 60 °C. Melt curve analysis was performed by (1) ramping to 95 °C at 1.6 °C/second and holding for 15 s, (2) ramping to 60 °C at 1.6 °C/second and holding for 1 min, and (3) ramping to 95 °C at 0.15 °C/s and holding for 15 s. Cycle thresholds and baselines were calculated using the automatic settings in the Quantstudio 12 K Flex software. Fold change was calculated using the 2^−ΔΔCT^ method [44].

### 2.7. Animals and Experimental Design

The immunodeficient Royal College of Surgeons (iRCS) rats breeding pair were as previously described [45]. Rats were group housed under specific pathogen-free conditions and had access to water and food ad libitum. At post-natal day 21 (±2 days), iRCS rats received 10 µL intravitreal injections of XVIVO10 (XV1), 3X concentrated XV1 (XV3), 20 µg/mL pigmented epithelial derived factor (PEDF, Bio-Techne, Minneapolis, MN), unconcentrated PRPE-SF (SF1), and concentrated SF1 (SF3) followed by general health and clinical ocular observations. This level of PEDF is equivalent to the concentrations found in SF3. The SF3 treatment group had a higher number of samples due to repeated studies performed over a 2-year period in which SF3 was tested against the various controls in agreement with the approved grant from the California Institute for Regenerative Medicine (TRAN1-11532). The data collected over this period were combined for analysis. Prior to each IVT injection, functional and structural assessment was performed by ERG and OCT, respectively. Animals received the second and third serial injections every 14 days at p35 (+2 days) and p49 (+2 days). Before each IVT injection, anesthesia was administered by abdominal injection of ketamine (37.5 mg/kg) and xylazine (5 mg/kg). Topical anesthesia and pupil dilation were induced by 0.5% proparacaine hydrochloride ophthalmic solution (Akorn, Inc., Lake Forest, IL, USA) and 2.5% phenylephrine hydrochloride and 0.5% tropicamide (Akorn, Inc.), respectively.

### 2.8. Electroretinogram (ERG) Evaluation

For ERG assessment, animals were placed in dark adaptation overnight the day before the functional testing. Under dark conditions and using a dim red light, animals were anesthetized as described above, along with application of pupil dilation and topical anesthesia eye drops. While the animal was under anesthesia, a heating table and monitor were used to monitor body temperature. Reference and ground electrodes were inserted into the infraorbital (malar) area and between the ears, respectively. Scotopic testing was conducted with flash stimuli intensities ranging from 0.1–25 candela (cd) and recorded from both eyes. Subsequently, rats were light-adapted and photopic testing was conducted with intensities from 0.01–25 cd (HMsERG Rodent System, OcuScience, NV, USA). ERG raw data were collected and evaluated.

### 2.9. Ocular Coherence Tomography (OCT) Evaluation

Imaging scanning was performed at the end of the ERG session. Using the Spectralis OCT (Heidelberg Engineering Inc., Franklin, MA, USA), the animal was placed over the animal-modified head support used for patients. Balanced salt solution (BSS) was applied regularly to moisten the cornea during the imaging acquisition. A full set of b-scans was acquired from both sides of the optic nerve. Each set of images included a high-resolution b-scan and a volume scan composed of 30 b-scans from the temporal and nasal sides of the optic nerve, from both eyes. Morphological analysis of the retina included a grading score based on the outer retina changes, as described in Supplementary Figure S1. By using the Heidelberg Eye Explorer software, the retinal thickness and ONL characteristics were evaluated. After the completion of the OCT imaging session, animals were injected with test articles.

### 2.10. Intravitreal Injection

Under sterile conditions, using a 30-gauge (G) needle, a scleral incision was performed behind the limbus in the temporal superior quadrant of the left eye. Subsequently, intraocular pressure was reduced by a puncture into the anterior chamber through the periphery of the cornea, also with a 30 G needle. Then, 10 µL of the tested article was injected through the scleral incision by using a 30 G blunt steel needle attached to a 50 µL Hamilton syringe. After the completion of the IVT injection, a clinical assessment of the posterior pole was carried out by clinical visualization through the surgical microscope. A self-sealed healing of the sclera and the conjunctiva was observed, and no sutures were needed. Topical application of antibiotic ointment was performed at the end of the procedure.

### 2.11. Euthanasia and Tissue Collection

At p60, rats were euthanized by intraperitoneal injection of 0.5 mL pentobarbitol sodium 390 mg and phenytoin sodium 50 mg (Euthasol; Virbac AH, Inc., Fort Worth, TX, USA) and their eyes were enucleated and fixed in Davidson’s solution. After 24 h of fixation, the Davidson’s solution was replaced with 70% ethanol and sent to the USC Ginsburg Institute for Biomedical Therapeutics Core for paraffin embedding, sectioning, and hematoxylin and eosin (H&E) staining. Anterior segment structures, including cornea, iris, and lens were removed and the posterior pole was exposed. The cut of the eye was through the optic nerve on its sagittal plane. After dissection, all eyes were embedded in paraffin and cut in a microtome starting from the center of the optic nerve. Serial sections of 5 µm in thickness were performed throughout the entire eyeball. Approximately 4 consecutive/serial retinal sections were placed on every glass slide.

### 2.12. Photoreceptor Counting

Surviving PRs were determined in rats treated with various test and control articles. Since injection of both the test and control articles were not localized to one area of the retina, as they were deployed within the vitreous cavity, slides representing the central area of the retina were selected for enumeration. Specifically, the slide for enumeration was chosen based on the presence of the optic nerve as a landmark. The preserved ONL cell numbers were enumerated from scanned images of the sections. Photoreceptor counting was initiated 1 mm superior or inferior to the optic nerve and continued for 1 mm. The “Nuclear V.09” algorithm (a nucleus counting algorithm) provided in the Aperio ScanScope CS microscope software was used to enumerate the cells. Two sections were counted for each animal and the results were averaged.

### 2.13. Immunofluorescence Staining

Eye tissue sections were first deparaffinized and rehydrated via immersion in a series of xylene, ethanol, and PBS solutions. Heat-induced epitope retrieval was performed using 1× citrate buffer (pH = 6.0) in a pressure cooker. Tissues sections were then permeabilized with 0.3% Triton X-100 in PBS for 10 min then blocked for 30 min with blocking buffer (PBS containing 2.5% normal goat serum (*v*/*v*)). Primary antibodies (Supplementary Table S3) were incubated overnight at 4 °C then incubated for 45 min at RT with secondary antibody diluted 1:500 with blocking buffer. Nuclear staining was performed using 1 µg/mL DAPI in PBS for 10 min at RT. Coverslips were mounted using VECTASHIELD Vibrance Antifade Mounting Medium (Vector Laboratories, Newark, CA, USA). Fluorescent images were taken on the Olympus BX43 microscope using a 40× objective. Immunofluorescence images were analyzed in ImageJ using 5 images per animal. ImageJ analysis was performed by manually selecting the retinal layers and using the DAPI channel to refine the nuclear layer regions of interest with a histogram threshold of 50–255 on an 8-bit scale. For 4HNE and MDA staining, automatic thresholding using the Phansalkar method was applied and then the percentage area per retinal layer was measured. This was similarly applied for PAD4 in the ONL and outer segments. For CitH3, histogram thresholds were set from 30–255 on an 8-bit scale and the percentage area within the DAPI mask was measured. CD68 cell counts were performed on images with threshold set from 30–255 on an 8-bit scale and particle analysis using 5–300 px^2^ size range for the entire image field.

### 2.14. Statistical Analysis

Statistical analysis was performed in GraphPad Prism 9 (Graphpad Software Inc., La Jolla, CA, USA). All graphs are plotted as mean ± standard error of the mean, unless otherwise noted in the figure legend. Appropriate statistical analyses were performed for each dataset, including unpaired *t*-tests for comparing two treatments. For comparing more than two groups, one-way ANOVA with Tukey’s correction, Welch’s ANOVA with Dunnett’s T3 correction, and Kruskal–Wallis with Dunn’s correction were performed based on the dataset. For genetic expression data, ddCT was compared using two-way ANOVA with Tukey’s correction. The statistical tests used in each individual analysis are indicated in the figure legends.

### 2.15. Study Approval

Regulatory approval for use of the hESC lines was obtained from the University of Southern California Stem Cell Research Oversight Committee (SCRO). Animal experiments were conducted in full accordance with University of Southern California Institutional Animal Care and Use Committee (IACUC)-approved protocols, National Institutes of Health Guide for the Care and Use of Laboratory Animals, and the ARVO Statement for the Use of Animals in Ophthalmic and Vision Research. The graphical abstract and Figure 1 were created with Biorender.com (accessed on 15 June 2023).

## 3. Results

### 3.1. PRPE-SF Increases fRPC Cellular Viability and Rhodopsin Expression

**Figure 2:**
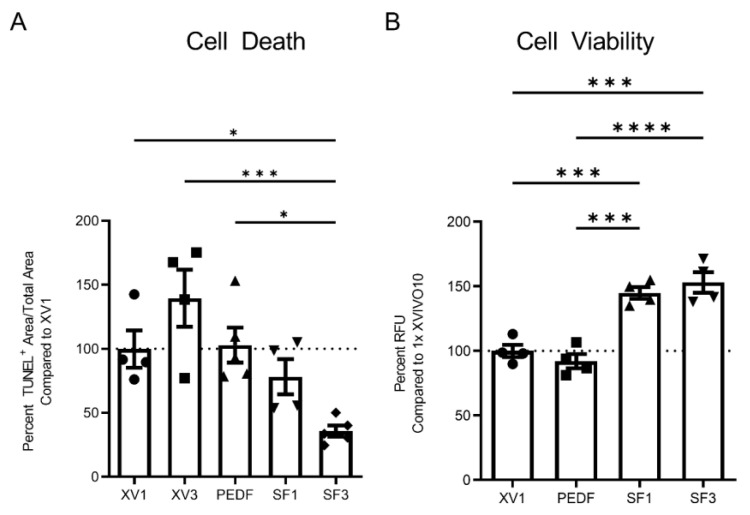
fRPCs treated with SF3 showed significant decrease in cell death compared with all other treatments as determined via the apoptosis TUNEL assay following 24 h incubation (*n* = 4 XV1, XV3, and SF1, *n* = 5 PEDF and SF3) (**A**). Both SF1 and SF3 showed significantly improved cell viability compared with XV1 and PEDF, assessed by resazurin metabolism following 24 h incubation (*n* = 4 each group) (**B**). Data represented as mean ± SEM. * p < 0.05, *** p < 0.001, **** p < 0.0001, one-way ANOVA with Tukey’s correction.

Cellular apoptosis and proliferation of fRPC were compared among XV1, XV3, 20 µg/mL PEDF, SF1, and SF3. PEDF was chosen as a control as it is highly concentrated in the mature RPE secretome and is known to promote retinal cell survival [26]. The concentration of PEDF used reflects the levels found in SF3 as measured using ELISA. TUNEL staining was used to quantify the number of apoptotic cells, and propidium iodide was used as the counterstain to measure total cell area. The apoptotic/total cell ratio was then normalized to XV1 (Figure 2A). The results showed that the SF3 treatment significantly reduced the apoptotic/total cell ratio, while no reduction was observed with other treatments, including 20 µg/mL PEDF. In addition, both SF1 and SF3 improved cellular viability compared with XV1 and PEDF, as determined by the resazurin assay (Figure 2B).

**Figure 3:**
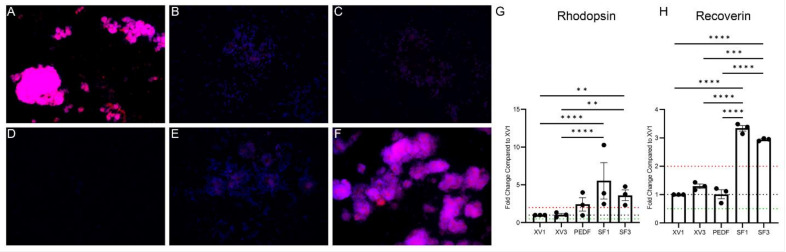
Rhodopsin and DAPI staining of fRPC immediately after isolation (**A**) and after 24-h incubation with XV1 (**B**), XV3 (**C**), PEDF (**D**), SF1 (**E**) and SF3 (**F**). RT-qPCR shows SF1 and SF3 induce rhodopsin (**G**) and recoverin (**H**) gene expression. Data represented as mean ± SEM. ** p < 0.01, *** p < 0.001, **** p < 0.0001, statistically analyzed ddCT values with one-way ANOVA with Tukey’s correction using data from *n* = 3 independent experiments with triplicate samples in each experiment.

The impact of SF1 and SF3 on photoreceptor survival was evaluated using rhodopsin expression in fRPCs, comparing XV1, XV3, and 20 µg/mL PEDF (Figure 3A–F). Freshly isolated fRPCs displayed intense rhodopsin immunofluorescent staining (Figure 3A), however, 24 h after treatment with XV1, XV3, PEDF, or SF1, the level of rhodopsin was minimally detectable (Figure 3B–E). In contrast, SF3-treated fRPC maintained high levels of rhodopsin staining (Figure 3F). Correspondingly, increased gene expression of rhodopsin and recoverin were observed for both SF1- and SF3-treated fRPC (*p* < 0.05) when compared with XV1 treatment (Figure 3G,H).

### 3.2. PRPE-SF Induces Gene Expression in fRPC

**Figure 4:**
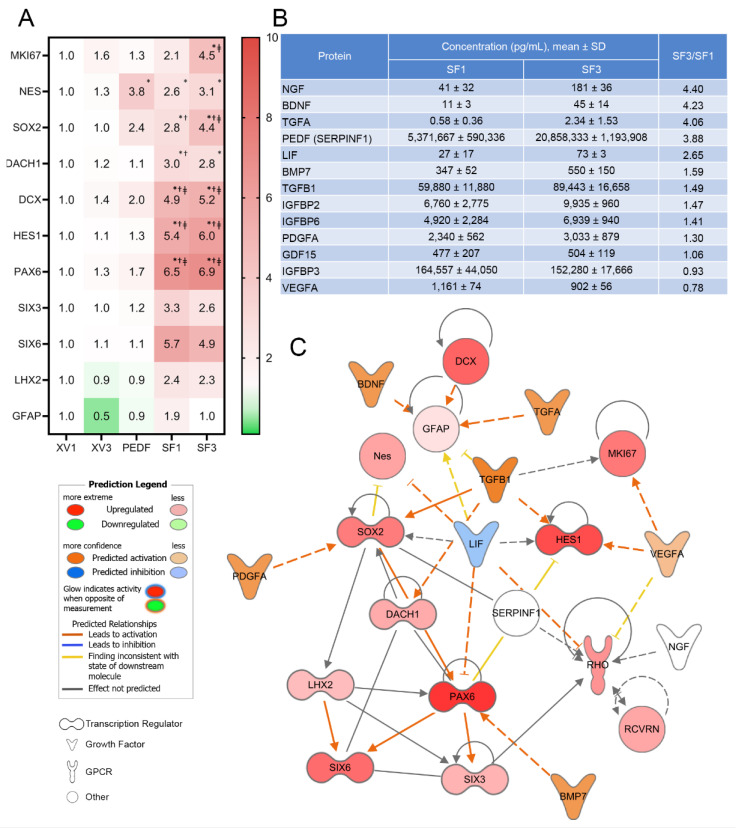
Mean fold change of fetal RPC gene expression after 24 h incubation compared with XV1 (**A**). RT-qPCR shows that SF1 and SF3 induce several eye-field transcription factor genes relating to proliferation and retinal development. Mean ± SD concentration of proteins in SF1 and SF3 determined by ELISA or microarray (**B**). Relationships between transcription factors (TFs), mature retinal cell markers, and RPE neurotrophic factors using IPA (**C**). TFs show a complex network of positive and negative regulations on each other, and PRPE-SF components additionally show both positive and negative regulations on TFs. Statistically analyzed ddCT values using two-way ANOVA and Tukey’s multiple comparisons of data from *n* = 3 independent experiments with triplicate samples in each experiment, no comparisons were performed for SIX3, SIX6, LHX2, and GFAP which had only *n* = 2 experiments. (* p < 0.05 compared to XV1, ^†^ *p* < 0.05 compared to XV3, ^ǂ^ *p* < 0.05 compared to PEDF.) Individual graphs can be viewed in Supplementary Figure S2. Networks generated with QIAGEN IPA (QIAGEN Inc., https://digitalinsights.qiagen.com/IPA, accessed on 16 March 2023).

To characterize the biological activities associated with SF1 and SF3, the impact of these treatments on fRPC was evaluated using targeted gene expression (Figure 4A, Supplementary Figure S2). Major constituents of SF1 and SF3, determined by microarray and ELISA, are highlighted in Figure 4B showing that NGF, BDNF, PEDF, LIF, TGFβ1, and BMP7 were all present and were enriched by our processing. SF1 and SF3 were able to upregulate progenitor cell proliferation, maturation, and differentiation-associated gene expression.

To further dissect the molecular interactions, an ingenuity pathway analysis (IPA) was undertaken. Factors such as PEDF, IGFBP3, and TGFβ1 were detected in high concentrations in both SF1 and SF3. TGFβ1, a pleiotropic factor, can upregulate SOX2, HES1, MKI67, and DACH1 (Figure 4A,C). SF1 and SF3 both upregulated MKI67 expression coinciding with the ability to promote fRPC viability. TGFβ1 can increase HES1 expression which regulates cell division, gliogenesis, and maintenance of tissue compartment boundaries [46]. Other properties ascribed to levels of TGFβ1 found in SF1 and SF3 include upregulation of DACH1, which has been shown to promote transition from progenitor cells to neuronal precursor cells achieved through cell cycle synchronization and interaction with cyclin D1 [47]. In addition, SF1 and SF3 were able to upregulate SOX2 expression by 2.8- to 4.4-fold, respectively, which can in turn trigger downstream upregulation of PAX6 and LHX2 expression. IPA also shows the contribution of BMP7 to increase expression of PAX6 where there were 6.5- and 6.9-fold increases compared with fRPC treated with XV1. LHX2, a central factor coordinating optic cup development, is required for BMP signaling and interacts with PAX6 to regulate SIX3 and SIX6 expression [48,49]. SF1 and SF3 upregulated DCX and NES, which are microtubule and cytoskeleton associated enzymes that play a role in the migration of progenitors and the structural orientation of retinal layers [50,51].

While SF1 and SF3 promote progenitor cell proliferation, maturation, and differentiation gene expression, they do not alter GFAP expression, astrocyte markers, or Müller development and reactivity [52]. IPA suggests that upregulation of the transcription factors is consistent with cells fated for the eye, proliferation of progenitor cells, and differentiation pathways [53,54,55]. While this is a targeted analysis, the relationships identified in IPA are evidence of critical components to monitor in future manufacturing development for a clinical grade product.

### 3.3. PRPE-SF Preserves Retinal Structure and Function in RCS Rats

**Figure 5:**
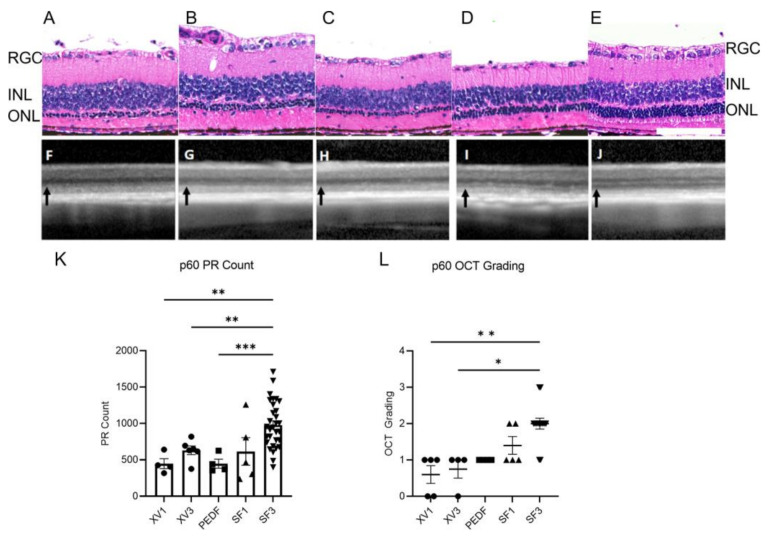
H&E and OCT images of p60 RCS retinas treated with XV1 (**A**,**F**), XV3 (**B**,**G**), PEDF (**C**,**H**), SF1 (**D**,**I**), and SF3 (**E**,**J**). H&E-stained retinas show outer nuclear layer (ONL) thickness correlating to OCT ONL (black arrow). The ONL was minimally detected in all treatment groups except for SF3 (**J**) of p60 iRCS retinas. Photoreceptor counts from H&E images ((**K**), XV1 *n* = 4, XV3 *n* = 6, PEDF *n* = 4, SF1 *n* = 5, SF3 *n* = 31) and OCT grading scores ((**L**), XV1 *n* = 5, XV3 *n* = 4, PEDF *n* = 4, SF1 *n* = 4, SF3 *n* = 14) show SF3 treatment significantly preserves the ONL compared with XV1 and XV3. Data represented as mean ± SEM, * p < 0.05, ** p < 0.01, *** p < 0.001, Photoreceptor counts: Welch’s ANOVA with Dunnett T3 correction; OCT grading: Kruskal–Wallis with Dunn’s correction. Scale bar is 100 µm.

The highest SF3/SF1 proteins are known to have antioxidant effects, including NGF, BDNF, PEDF, and LIF [56,57,58,59], suggesting that SF3 would have greater antioxidant effects and efficacy in models of retinal degeneration. Experimental media were administered via intravitreal injection (IVT) on p21, p35, and p49 in athymic RCS (iRCS) rats, and on p60, eyes were collected and subjected to molecular dissection. H&E staining revealed photoreceptor preservation in iRCS rats treated with SF3, with a statistically significant increase in photoreceptor nuclei counts when compared with XV1, XV3, and 20 µg/mL PEDF (Figure 5A–E,K). Furthermore, photoreceptor inner segments appeared to be better organized in SF3-treated retinas compared with other treatments. This portion of the photoreceptor is where protein synthesis machinery and mitochondria are localized. Photoreceptor preservation was also evaluated non-invasively throughout the study using OCT and confirmed with histological findings on p60 (Figure 5F–J,L).

**Figure 6:**
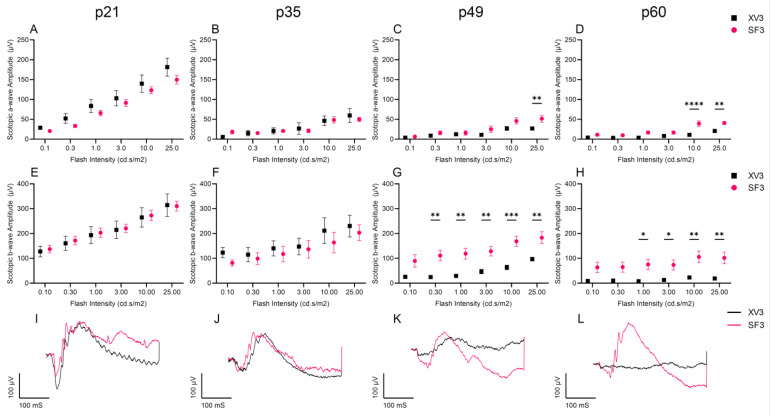
Scotopic ERGs of XV3- and SF3-treated eyes were measured using flash intensities of 0.1–25 cd∙s/m^2^. Scotopic a-wave (**A**–**D**) and b-wave (**E**–**H**) amplitudes were measured prior to intravitreal injection at p21–p60. Scotopic a-wave and b-wave were both significantly higher in SF3-treated rats at p49 and p60. A representative 25 cd∙s/m^2^ scotopic ERG is shown for each timepoint (**I**–**L**). Data represented as mean ± SEM, * p < 0.05, ** p < 0.01, *** p < 0.001, **** p < 0.0001, two-way ANOVA with Bonferroni’s correction (p21: XV3 *n* = 8, SF3 *n* = 16; p35: *n* = 4 both groups; p49: *n* = 4 both groups; p60: XV3 *n* = 6, SF3 *n* = 10).

**Figure 7:**
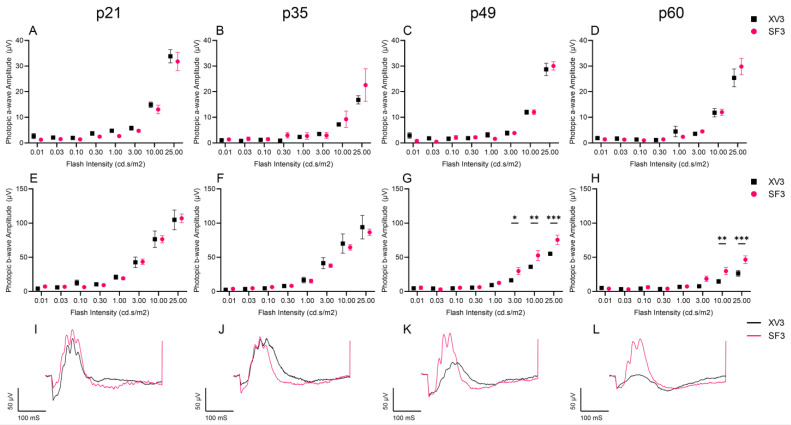
Photopic ERGs of XV3- and SF3-treated eyes were measured using flash intensities of 0.01–25 cd∙s/m^2^. Photopic a-wave (**A**–**D**) and b-wave (**E**–**H**) amplitudes were measured prior to intravitreal injection at p21-p60. Photopic b-wave amplitudes were significantly higher in SF3 treated rats at p49 and p60. A representative 25 cd∙s/m^2^ photopic ERG is shown for each timepoint (**I**–**L**). Data represented as mean ± SEM, * p < 0.05, ** p < 0.01, *** p < 0.001, two-way ANOVA with Bonferroni’s correction (p21: XV3 *n* = 8, SF3 *n* = 16; p35: *n* = 4 both groups; p49: *n* = 4 both groups; p60: XV3 *n* = 6, SF3 *n* = 10).

To investigate whether photoreceptor preservation translates to preservation of retinal function, scotopic and photopic ERG was performed prior to IVT injection at p21, p35, p49, and p60 in the XV3 and SF3 groups. The scotopic a-wave and b-wave amplitudes, used to measure rod photoreceptor response, showed significantly preserved retinal function in SF3-treated retinas at p49 and p60 (Figure 6). Additionally, the photopic b-wave amplitudes were significantly higher in SF3-treated retinas than XV3-treated retinas at p49 and p60, while the a-wave amplitudes were negligible and did not decline over time (Figure 7). Together, this demonstrates the effectiveness of SF3 in preserving retinal function.

### 3.4. PRPE-SF Reduces Reactive Oxygen Species and Reactive Glial Activation

**Figure 8:**
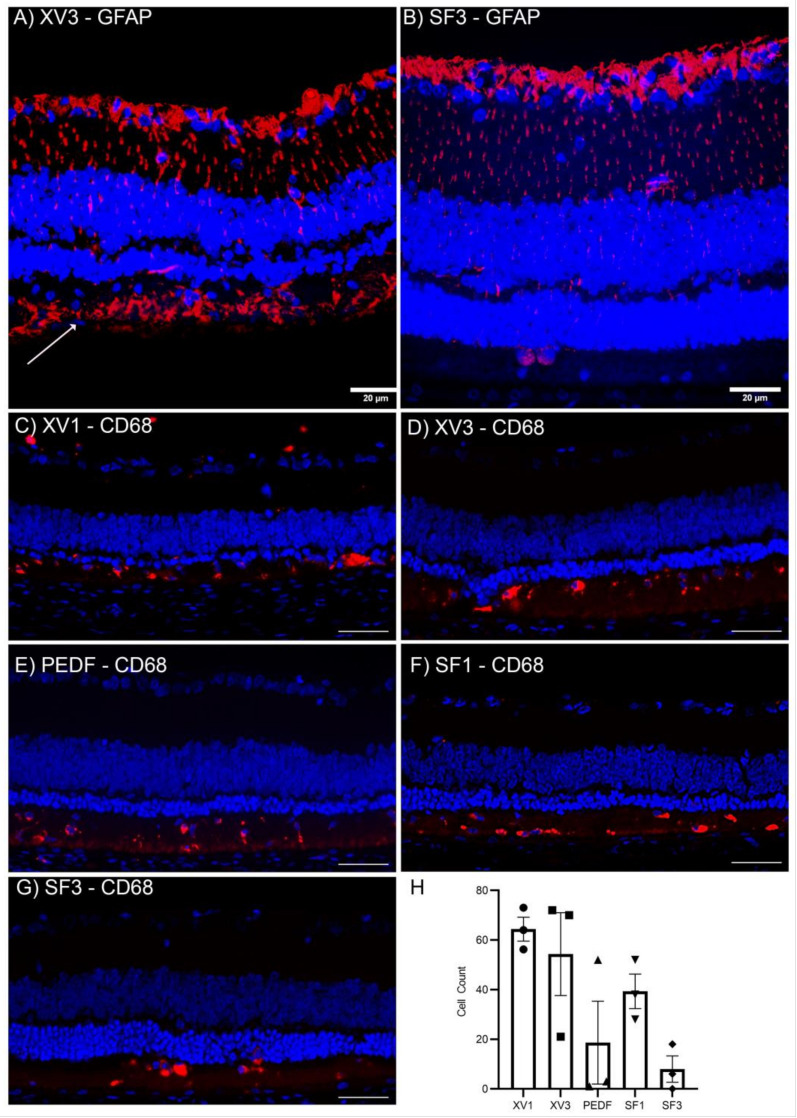
Immunofluorescence images of treated p60 iRCS rat retinas. XV3-treated retinas displayed significant gliosis as marked by GFAP staining throughout the retina and below the ONL ((**A**), white arrow) while SF3 retinas demonstrated reduced GFAP staining (**B**). Anti-CD68 immunofluorescence staining of p60 iRCS retinas (**C**–**G**). PEDF and SF3 show non-significantly reduced CD68+ cells ((**H**) *n* = 3 each group). Blue: DAPI. Red: GFAP (**A**,**B**) or CD68 (**C**–**G**). Scale bars are 20 µm (**A**,**B**) and 100 µm (**C**–**G**).

As shown above, SF3 was able to preserve photoreceptor and retina structure while SF1 did not. Immunostaining of RCS retinas as well as in vitro experiments in ARPE-19 cells further demonstrated that the increased efficacy of SF3 is linked to reduced Müller and microglial cell activation and reduced oxidative stress markers, which are also known to induce inflammation [2]. The in vivo administration of SF3 reduced the activation of Müller and microglial cells, as indicated by decreased levels of GFAP (Figure 8A,B) and CD68 (Figure 8C–G) staining, respectively. XV3-treated iRCS retinas showed evidence of gliosis as indicated by strong GFAP staining as well as subretinal glial membranes below the outer nuclear layer (ONL), which was reduced or not present in SF3-treated retinas. GFAP is commonly upregulated in retinal degeneration models, where the reduction in GFAP may indicate reduced gliosis [60]. XV1- and XV3-treated iRCS retinas showed significant CD68+ staining in the outer segments, while retinas treated with PEDF and SF3 demonstrated a dramatic reduction in CD68+ cells. However, the sample size was low and the reduction in CD68+ cells was not found to be statistically significant.

**Figure 9:**
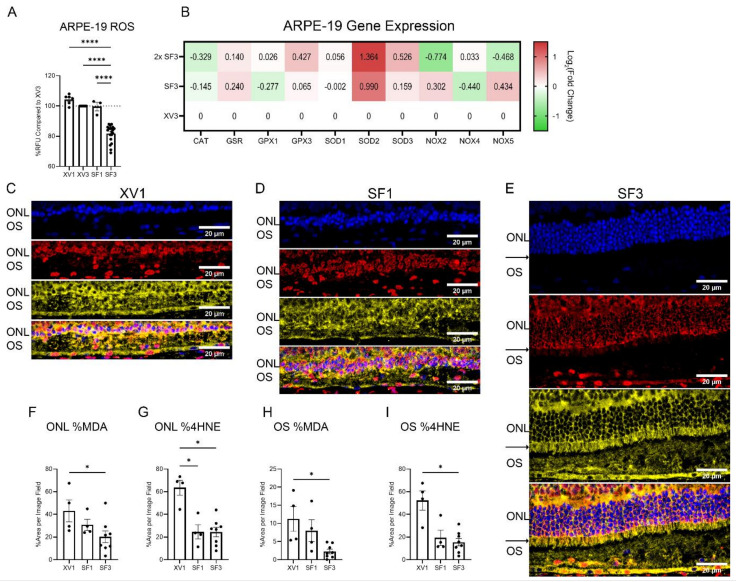
Effects of PRPE-SF on retinal reactive oxygen species. In vitro, SF3 significantly reduces DCFDA oxidation, a non-specific marker for reactive oxygen species, in ARPE−19 cells following 24 h incubation compared with all other treatments (**A**) (one-way ANOVA with Tukey’s correction using data from 17 independent experiments with triplicate samples in each experiment). It was observed that 2x SF3 increases significantly increased SOD2 expression following 24 h of treatment (mean Log_2_(Fold Change) > 1, duplicates) (**B**). Immunofluorescence images of p60 iRCS rats treated with XV1 ((**C**) *n* = 4), SF1 ((**D**) *n* = 4), and SF3 ((**E**) *n* = 8). Malondialdehyde (MDA; red) shows strong nuclear staining while 4-hydroxynonenal (4HNE; yellow) is primarily non-nuclear. Due to preservation, SF3 images appear larger, but each image shows the outer nuclear layer (ONL) and outer segment (OS) at the same magnification. SF3 treatment shows preservation of inner segments (black arrows in (**E)**) with strong staining for 4HNE. SF3 shows reduced photoreceptor nuclei staining of MDA and reduced ONL and OS staining of 4HNE. ImageJ analysis shows a significant decrease in both MDA and 4HNE percent area in the ONL and OS (Kruskal–Wallis with Dunn’s multiple comparisons) (**F**–**I**). * p < 0.05, **** p < 0.0001. Blue: DAPI. Red: MDA. Yellow: 4HNE. Scale bar is 20 µm.

The effects of SF3 treatment on the levels of ROS were also evaluated (Figure 9). In ARPE-19 cells, DCFDA was used to measure the cytoplasmic levels of ROS following 24 h incubation of XV1, XV3, PEDF, SF1, and SF3. SF1 had little effect on ROS levels, while concentrated SF3 was found to reduce ROS levels by approximately 20–30% compared with XV3 (Figure 9A). ARPE-19 treated with decreased dilutions of SF3 showed alteration in genes regulating ROS production and elimination (Figure 9B). Few to no changes in NADPH-oxidase (NOX) expression of multiple isoforms were found. However, increased SOD2 expression was seen. While SOD3 was also increased in a concentration-dependent manner, the expression changes did not reach significance. In addition, the glutathione pathway was also probed, and changes were not found.

To determine whether these in vitro and molecular findings correspond to in vivo studies, the effects of SF3 on oxidative products, such as malondialdehyde (MDA) and 4-hydroxy-2-nonenal (4HNE), were also studied in treated iRCS retinas. Results showed that iRCS rats treated with XV1 or SF1 had high levels of MDA and 4HNE in all retinal layers, while iRCS rats treated with SF3 showed reduced retinal levels of MDA and 4HNE, particularly in the ONL and outer segments (OS) (Figure 9C–I). The areas of MDA and 4HNE were measured as a percentage of the respective layer to account for increased retinal thickness in SF3 retinas. Staining showed that MDA had a stronger nuclear staining, while 4HNE appeared to be cytoplasmic and extracellular, suggesting separate roles for each lipid peroxide in retinal pathologies. Furthermore, SF3-treated retinas showed 4HNE localized within preserved inner segments, which links the ROS produced by photoreceptor mitochondria to the 4HNE staining of the inner segments. A longitudinal study was performed to verify that both MDA and 4HNE increase with the age of the iRCS rat (Supplementary Figure S3). The results showed that MDA showed significant increased expression in the ONL at p49 and p60, while 4HNE showed pan-retinal increases with the strongest increases in the outer plexiform layer, ONL, OS, and RPE layers.

### 3.5. PRPE-SF Reduces NETosis Markers PAD4 and CitH3

**Figure 10:**
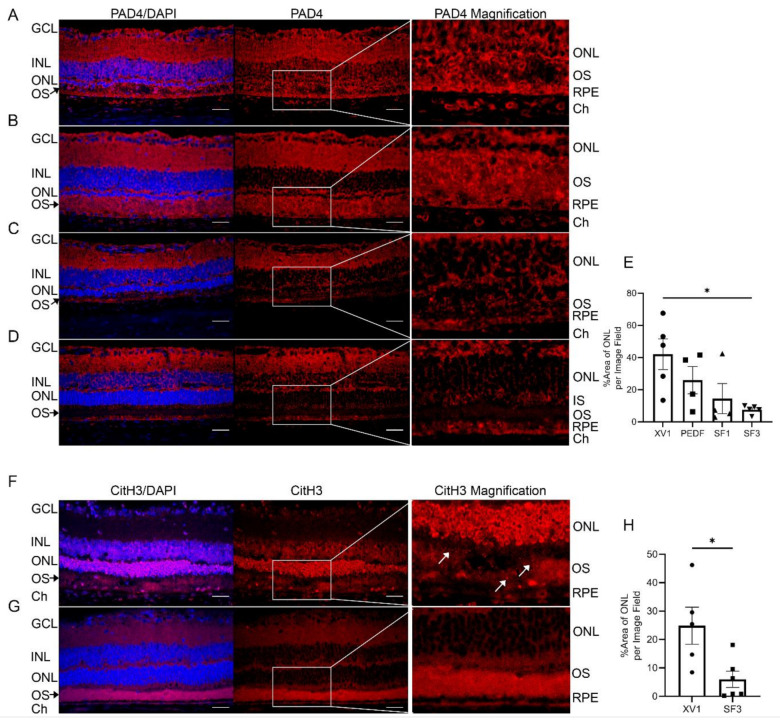
PAD4 immunofluorescence images of p60 iRCS rats treated with XV1 ((**A**), *n* = 5), PEDF ((**B**), *n* = 4), SF1 ((**C**), *n* = 4), and SF3 ((**D**), *n* = 5). PAD4 shows pan-retinal staining with strong staining in the ganglion cell layer (GCL), inner nuclear layer (INL), outer nuclear layer (ONL), outer segments (OS), retinal pigment epithelium (RPE), and choroid (Ch) in XV1- and PEDF-treated retinas. SF1- and SF3-treated retinas show reduced staining intensity; ImageJ analysis shows SF3-treated retinas have significantly reduced PAD4 percent area of ONL compared with XV1 (**E**). (* *p* < 0.05, one-way ANOVA with Tukey’s multiple comparisons). Similar to 4HNE, light PAD4 staining is seen in the preserved inner segment region (IS) of SF3-treated retinas (D). Citrullinated histone H3 (CitH3) immunofluorescence images of iRCS rats at p60 treated with XV1 ((**F**), *n* = 5) and SF3 ((**G**), *n* = 6). XV1-treated retinas show GCL, Ch, and extensive ONL staining with punctate debris in the OS (white arrows). ImageJ analysis of ONL CitH3 shows SF3-treated retinas have reduced ONL CitH3 expression compared with XV1 (**H**). (* *p* < 0.05, unpaired *t*-test). Blue: DAPI. Red: PAD4 or CitH3. Scale bars are 20 µm.

In recent years, sterile neutrophil extracellular trap (NET) formation has emerged as a crucial factor in retinal disease and is known to result from increased oxidative stress and play a role in retinal autoimmunity [61]. Since increased ROS production can lead to PAD4 activation [62], the expression of PAD4 was evaluated in retinas of iRCS rats treated with XV1, PEDF, SF1, and SF3 at p60 (Figure 10A–E). XV1- and PEDF-treated retinas revealed high PAD4 expression present in all retinal layers, including the inner nuclear layer (INL), ONL, and OS. However, retinas treated with SF3 exhibited reduced PAD4 expression, particularly in the ONL and OS. These findings indicate the protective effect of SF3 in reducing PAD4 expression. In addition, the presence of PAD4 in the choroid of retinas treated with XV1 and PEDF highlights the presence of both intra- and extra-retinal sources of PAD4.

Retinas were also analyzed for citrullinated histones (CitH3) which are produced through PAD4-mediated deimination. Results showed that retinas treated with XV1 had high levels of CitH3 in various retinal layers including the ganglion cell layer (GCL), INL, ONL, RPE, and choroid. However, retinas treated with SF3 demonstrated significantly reduced levels of CitH3 (Figure 10F–H). These findings provide evidence that reduction in PAD4 expression and citrullination correspond with delayed retinal degeneration, highlighting the potential therapeutic benefits of SF3 in retinal disease pertaining to autoimmunity.

## 4. Discussion

Therapeutic options for retinal degenerative diseases are extremely limited, whether for AMD or genetic conditions such as retinitis pigmentosa. However, stem cell implantation has emerged as a promising therapeutic approach for directly replacing atrophic cells [11]. Previously, the implantation of hESC-RPE showed that these cells were able to re-epithelialize the dystrophic areas and extend preservation beyond the borders of the implant [16,44]. This effect may be associated with the secretome elaborated by the implanted RPE that is capable of maintaining ocular function, coinciding with reduced ocular oxidative stress and inflammation. The key aspects of PRPE-SF are: (1) PRPE-SF utilizes the biomimetic membrane to induce human ESC-RPE to polarize and mature, thus modulating the secretome towards a protective phenotype [63,64]; (2) PRPE-SF does not use animal serum, which will aid in clinical development to avoid immunogenicity; and (3) PRPE-SF is a combination of neuroprotective factors which can address multiple pathological mechanisms of retinal degeneration. This study examined the therapeutic potential of SF3 for treatment of retinal degenerative conditions by promoting neuronal survival while mitigating oxidative stress and inflammation.

The RPE is a source of neuroprotective factors, however, purified factors when given alone have failed in clinical evaluation. Previous research demonstrated that conditioned medium derived from rat RPE was able to promote rat RPC survival and differentiation [30,31,65]. Similarly, human-derived RPE-conditioned medium was able to reduced cellular death of porcine retinal explants [18]. Previously, we expanded on these findings showing that hESC-derived RPE-conditioned medium can promote fRPC proliferation while inhibiting cell death [63]. Here, we further showed that PRPE-SF treatment of fRPCs was able to upregulate genes associated with progenitor cell proliferation and multipotency through upregulation of MKI67, SOX2, and PAX6. Furthermore, PRPE-SF promoted eye fate determinants (DACH1 and LHX2) and promoted neuronal migration genes (NES and DCX). The enriched secretome was able to upregulate photoreceptor developmental genes, rhodopsin and recoverin, but did not affect glial genes such as GFAP. These findings are consistent with studies where RPE-conditioned media preferentially differentiated rat retinal explants and immortalized RPCs into photoreceptors [33,66].

Upregulation of these genes suggests that PRPE-SF may induce proliferation and differentiation of latent progenitor cells towards neuronal fates, which is being explored in current studies. According to upstream analysis and predictive modeling in IPA, there is a direct relationship between PRPE neurotrophic factors and the effected transcription factor genes in our fRPC dataset. However, SF3 showed a stronger effect on reduced cell death and preserved rhodopsin staining, potentially through protective mechanisms rather than proliferation. Several growth factors in SF1 were proportionally higher in SF3. Proteins with high SF3/SF1 ratios, including NGF, BDNF, PEDF, and LIF, are known to have protective effects for neurodegenerative disorders and probably contribute to blockage of pro-apoptotic pathways [58,59,67]. NGF and PEDF are particularly interesting as these trophic factors have been individually investigated in clinical trials for retinal conditions, including NGF for retinitis pigmentosa, cystoid macular edema, and glaucoma, and PEDF for macular degeneration [68,69,70].

Previous research has demonstrated that RPE cells secrete a mixture of anti-angiogenic and pro-angiogenic factors, and the balance is shifted towards pro-angiogenic factors in oxidatively stressed RPE cells [36]. While SF3 contains a mixture of these factors, we hypothesized that the unstressed, healthy RPE cells produce these factors at a concentration, which can restore this imbalance and promote retinal preservation. A link between these neurotropic factors and oxidative stress led us to interrogate the impact of SF3 in iRCS. IVT administration of SF3 starting on p21 and given bi-weekly till p60 was able to significantly preserve photoreceptors. More importantly, photoreceptor preservation corresponded with the preservation of scotopic a-wave and scotopic and photopic b-wave amplitudes, suggesting that SF3 can also ameliorate and delay decline in retinal function. This study also suggests that antioxidant mechanisms of SF3 contribute to retinal preservation. ARPE-19 treated with SF3 was consistently able to reduce cytoplasmic ROS by 20%. Amongst the ROS-generating and -eliminating genes investigated, SF3 significantly increased SOD2 and marginally increased SOD3 gene expression in ARPE-19. Increased SOD2 and SOD3 expression would increase the rate of superoxide elimination resulting in the observed ROS reduction. These findings coincide with reduction of ROS in vivo, where reduced levels of MDA and 4NHE were seen in SF3-treated iRCS retinas compared with XV1. The MDA reduction was particularly dramatic in the ONL and OS, suggesting that SF3 reduced oxidative stress in areas of photoreceptor degeneration and subsequent microglia activation and translocation. The level of 4HNE, a cytotoxic byproduct of arachidonic acid metabolism, was also lower in the ONL and OS layers of iRCS treated with SF3. Both peroxides form protein and DNA adducts that can induce additional inflammatory responses and perpetuate chronic inflammation as seen in retinal degeneration [2]. Additionally, MDA accumulation has been linked to RPE dysfunction and VEGF secretion in AMD [71]. Microglia and macrophage cells infiltrate in response to these damage-associated molecular patterns (DAMPs) released by damaged photoreceptors in an effort to clear cellular debris and detoxify the local microenvironment [72]. The reduction in ROS and oxidative byproducts may partially explain why SF3 treatment was associated with reduced infiltration of CD68+ cells into the retina.

In this study, ROS elevation was present in iRCS retinas corresponding to PAD4 activation, which is consistent with studies showing elevated intraretinal citrullination in human AMD donor eyes [73]. SF3 was able to reduce ONL and OS PAD4 staining coinciding with reduced CitH3. Additionally, PAD4 and CitH3 choroidal staining was observed in XV1-treated retinas and reduced with SF3. Choroidal neutrophils have been implicated in AMD and the degeneration of the RPE barrier [74], which suggests retinal remodeling associated with sterile inflammation initiated by aberrant oxidative stress and photoreceptor cell debris. Binet et al. suggested that vascular endothelial cells in diseased blood vessels engage in molecular pathways similar to those in aging. Cellular damage ultimately lead to cellular senescence-associated cytokine secretion, recruiting neutrophils that can subsequently trigger NETs [75]. While Binet et al. proposed that NETs can facilitate elimination of diseased senescent vasculature as a protective mechanism, chronic NETosis overactivation probably results in further destruction of the retinal vasculature. This is corroborated in studies demonstrating that NETosis contributes to the chronic inflammatory microenvironment of colorectal tumors [76]. In addition to infiltrating neutrophils in retinal vasculature, Müller cells may be responsible for PAD4 and CitH3 expression in the ONL and OS due to their known expression of PAD4 during reactive gliosis [77]. While this mechanism requires further exploration, SF3 treatment can ameliorate PAD4 and CitH3 staining in iRCS, which is paralleled by the reduction of 4HNE and MDA in treated eyes and retinal preservation.

This dataset shows clear concentration-dependent relationships with photoreceptor preservation using both in vitro and in vivo models. Because SF1 and SF3 had similar effects on viability and proliferation, the antioxidant and anti-inflammatory effects of SF3 are likely to be more important than the mobilization of progenitor cells for prevention of retinal degeneration. Additionally, the preservation of ERG at only p49 and p60 may be an additional indicator of the primary mechanism of PRPE-SF. The RCS rat is a Mertk mutant model of retinal degeneration, in which the primary cause of degeneration is the failure to phagocytose photoreceptor outer segments which require daily renewal [78]. Photoreceptors begin to die in response to accumulating debris and the oxidative microenvironment. As shown with MDA and 4HNE staining, significant staining is not observed until p49, and this accumulating oxidative stress accelerates the degeneration of the retina. Together, this suggests that the p49 ERG preservation is due to the protective effect of PRPE-SF against cytotoxic oxidation.

Our research is primarily limited by the availability of adequate models of retinal degeneration for AMD, which is the most prevalent cause of human retinal disease. However, we chose to utilize the RCS model as it is well characterized and has been utilized in the development of the CPCB-RPE1 implant currently under clinical investigation [12]. The RCS rat model is an aggressive model of retinal degeneration, making long-term efficacy studies untenable. In general, the predictivity of animal studies for human translation is low as many developed therapeutics fail in clinical settings [79]. As such, long-term efficacy studies are best evaluated in clinical studies and a stringent safety profile is developed in pre-clinical stages. We have conducted our work using SF3 stored for roughly a year, indicating the stability of the product when stored properly. In addition, we are performing scalability and long-term safety studies to support advancement to clinical stages. The delivery route and frequency of SF3 is also agreeable, with future clinical applications in which monthly IVT injections are common in the treatment of human retinal pathologies, although we utilized biweekly injections due to the aggressive nature of the RCS retinal degeneration. Further investigation into the underlying molecular mechanisms of retinal degeneration is still ongoing and will better support our analysis. However, we have demonstrated that SF3 affects key mechanisms related to retinal degeneration, such as oxidative stress and inflammation. We have also identified the potential pathological change in citrullination of the photoreceptor nuclei, which has not been observed in prior research. While this requires further elucidation, we are excited by the potential of a new therapeutic target for retinal dystrophies. Lastly, this study was conducted in a specific model of retinal degeneration, but due to the general antioxidant and anti-inflammatory effects, we are hopeful that it can be generalized to other age-related and neurodegenerative conditions.

## 5. Conclusions

In conclusion, our study underscores the therapeutic potential of PRPE-SF, particularly SF3, in treating retinal degeneration. The data reveals that SF3, derived from PRPE cells, can (1) hinder retinal cell apoptosis, (2) diminish cellular ROS, and (3) alleviate ocular oxidative and inflammatory stress in the retinopathy of the RCS model. Our IPA predictive modeling results reveal a strong relationship between PRPE-SF and the affected genes in this study. Nonetheless, comprehensive characterization of PRPE-SF is warranted to satisfy regulatory requirements and to pinpoint the pivotal active component(s) responsible for mitigating the hostile microenvironment in degenerative models will enhance the reproducibility and effectiveness of PRPE-SF products.

Crucially, PRPE-SF exhibited the preservation of retinal structure and function, as assessed by clinically relevant methodologies. The development of products such as PRPE-SF represents an innovative frontier, previously inaccessible due to technological constraints. However, with the recent advancements in stem cell technology, PRPE-SF emerges as a promising candidate for addressing retinal pathologies, such as dAMD and retinitis pigmentosa. This development could represent a significant leap towards effective therapeutics for these debilitating conditions and may set a precedent for treating other age-related and neurodegenerative diseases.

## Data Availability

The data presented in this study are available on request from the corresponding author.

## Supplementary Materials

The following supporting information can be downloaded at: https://www.mdpi.com/article/10.3390/cells12131689/s1.
