# Supplementary material for: Polarized RPE Secretome Preserves Photoreceptors in Retinal Dystrophic RCS Rats
Source: Cells. 2023 Jun 22;12(13):1689. doi: 10.3390/cells12131689 (PMC10340490; doi:10.3390/cells12131689)
Supplement: Supplementary file 1 [file cells-12-01689-s001.zip › cells-2459687-supplementary.pdf]

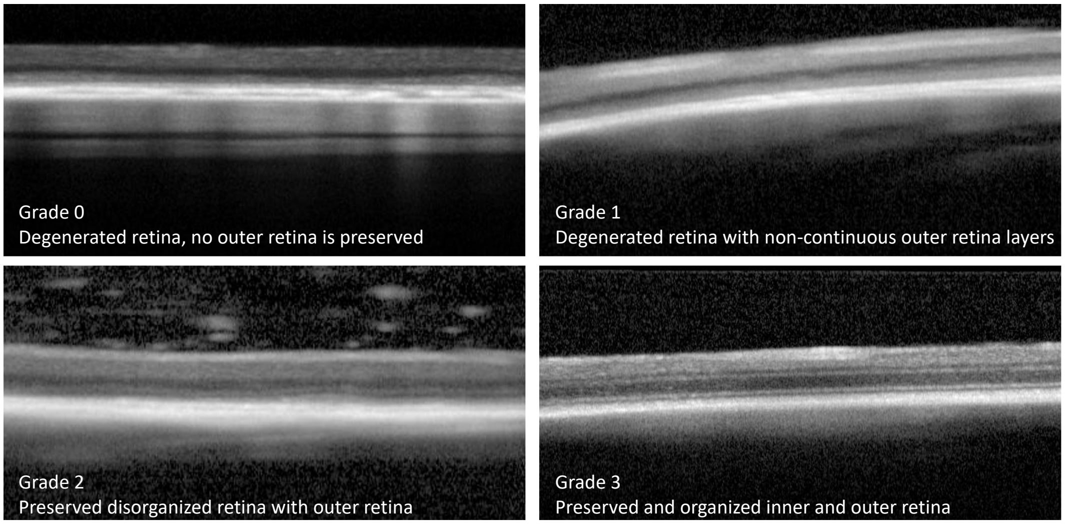

Supplemental Figure S1. Grading Score System by OCT. Grading system evaluated morphological changes at the outer retina at the end of the study.

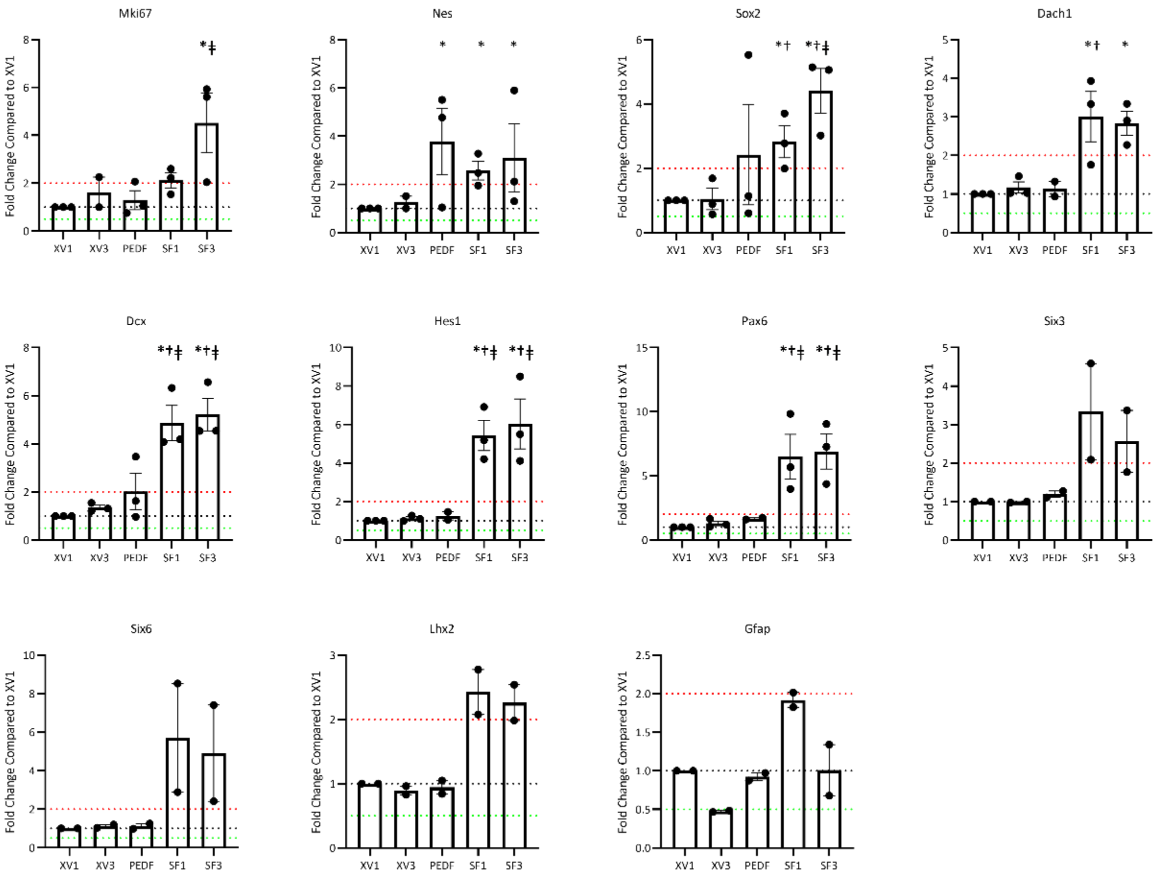

Supplemental Figure S2. Mean ± SEM Fold change of fetal RPC genetic expression after 24-hour incubation compared to XV1. RT-qPCR shows SF1 and SF3 induce several transcription factor genes relating to proliferation and retinal development. Statistics analyzed ddCT values with two-way ANOVA and Tukey's multiple comparisons using data from n=3 independent experiments with triplicate samples in each experiment, no comparisons were performed for Six3, Six6, Lhx2, and Gfap which only had n=2 experiments. *(*P<0.05 compared to XV1, †P<0.05 compared to XV3, ‡P<0.05 compared to PEDF).*

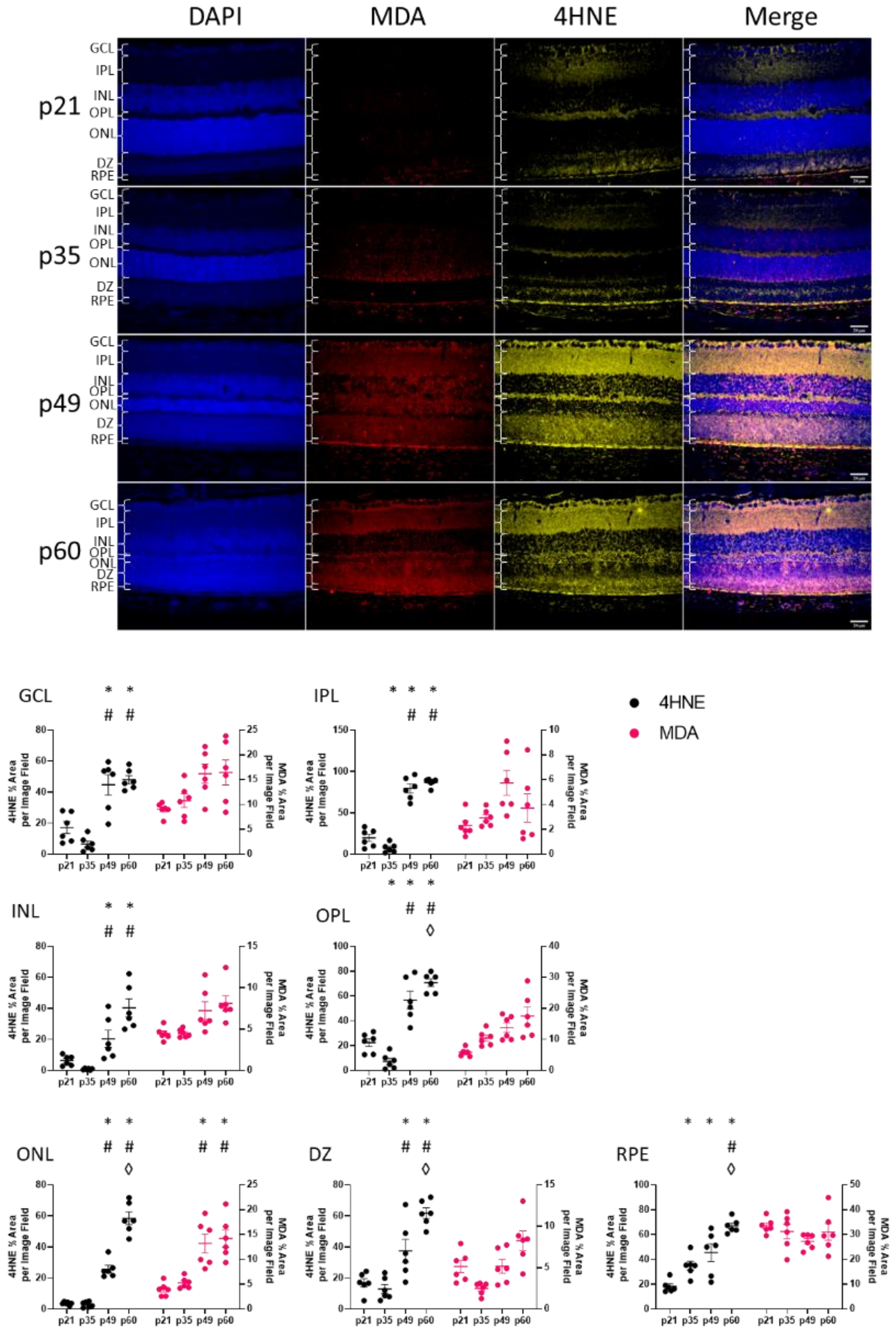

Supplemental Figure S3. Immunofluorescence staining and quantitation of 4HNE and MDA in RCS retinas throughout retinal degeneration. Data was analyzed using two-way ANOVA and Tukey's multiple comparison, n=6 each group. 4HNE shows increased staining in all retinal layers at p49 and p60 compared to p21 and increased intensity at p60 in the OPL, ONL, DZ, and RPE layers compared to p49. MDA showed significant increases at p49 and p60 in the ONL only. Comparisons between age with same immune status: *P<0.05, compared with P21; #P<0.05, compared with P30; ◊P<0.05, compared with P45. DAPI (blue), MDA (red), 4HNE (yellow). Scale bar is 20 μm.

Supplemental Table S1. Sigma-Aldrich Primers for fRPCs

Species	Gene	Gene ID	Primer Pair
Human	ACTB	60	1
Human	DACH1	1602	1
Human	DCX	1641	1
Human	HES1	3280	1
Human	PAX6	5080	1
Human	SIX3	6496	1
Human	SIX6	4990	1
Human	SOX2	6657	1
Human	MKI67	4288	1
Human	NES	10763	1
Human	LHX2	9355	1
Human	GFAP	2670	1

Supplemental Table S2. ARPE-19 Human Primers

Gene	Forward (5′-3′)	Reverse (5′-3′)
CAT	GAGGTTGAACAGATAGCCTTCG	GCGGTGAGTGTCAGGATAGG
GSR	CACAATAGAGGTCAGTGGGAAA	AAATCCATCGCTGGTTATTCC
GPX1	GCAACCAGTTTGGGCATCAG	CGTTCACCTCGCACTTCTC
GPX3	GCAGTATGCTGGCAAATACG	CCAGAATGACCAGACCGAAT
SOD1	GCAGATGACTTGGGCAAAGG	TGGGCGATCCCAATTACACC
SOD2	GCACTAGCAGCATGTTGAGC	AGATACCCAAAACCGGAGC
SOD3	CTACTGTGTTCCTGCCTGCTC	ACATGTCTCGGATCCACTCC
CYBB (NOX2)	AACGAATTGTACGTGGGCAGA	GAGGGTTTCCAGCAAACTGAG
NOX4	GCATGGCTGTGTCCTGGA	GAGCCAGATGAACAGGCAGA
NOX5	CTATTGGACTCACCTGTCCTACC	GGAAAAACAAGATTCCAGGCAC

Supplemental Table S3. Antibodies

Antibody	Host	Dilution	Catalog	Manufacturer
Rhodopsin	Mouse	1:400	ab3267	Abcam
GFAP	Rabbit	1:500	Z0334	Dako
CD68	Mouse	1:100	mca341r	Bio-Rad
PAD4	Rabbit	1:100	17373-1-AP	Proteintech
CitH3	Rabbit	1:1000	ab5103	Abcam
4HNE	Mouse	1:500	MAB3249	R&D Systems
MDA	Rabbit	1:1000	ab27642	Abcam
Mouse Secondary	Goat	1:500	A32723	Thermo Fisher Scientific
Rabbit Secondary	Goat	1:500	A11037	Thermo Fisher Scientific
